# Triglycerides to High-Density Lipoprotein Cholesterol Ratio Predicts Chronic Renal Disease in Patients without Diabetes Mellitus (STELLA Study)

**DOI:** 10.3390/jcdd7030028

**Published:** 2020-08-01

**Authors:** Vaia D. Raikou, Despina Kyriaki, Sotiris Gavriil

**Affiliations:** 1. Department of Nephrology, Doctors’ Hospital, 26 Kefallinias, Athens 11257, Greece; 2 Department of Nuclear Medicine, General Hospital “LAΪKO”, Athens 11527, Greece; dkyriaki@gmail.com; 3 Department of of Bariatric Surgery, Doctors’ Hospital, 26 Kefallinias, Athens 11257, Greece; sotiris@sgavriil.gr

**Keywords:** TG/HDL-C ratio, estimated pulse wave velocity, albuminuria, renal disease, hypertension

## Abstract

Background: The triglycerides to high-density lipoprotein cholesterol (TG/HDL-C) ratio has been included in the potential indices for atherosclerosis in chronic kidney disease (CKD). In this study, we addressed the role of the TG/HDL-C ratio on CKD prediction defined by both classified estimated glomerular filtration rate (eGFR) and classified urinary albumin-to-creatinine ratio (UACR) in non-diabetic participants. Methods: One hundred and eighty-three subjects with a mean age 67.3 ± 15.6 years old were included. Our participants were classified in both eGFR and UACR categories according to the Kidney Disease Improving Global Outcomes 2012 criteria. Estimated pulse wave velocity (ePWV) was calculated using an equation from age and mean blood pressure. The TG/HDL-C ratio was calculated. X2 tests and adjusted models were applied using confounders. Results: The TG/HDL-C ratio was inversely associated with eGFR and positively with both UACR and ePWV. We divided our patients in two groups according to the found ROC curve of the TG/HDL-C ratio cut-off point, either with an eGFR of less or more than 60 mL/min/1.73 m^2^. X^2^ tests showed significant association between the high TG/HDL-C ratio and classified eGFR, and classified UACR and hypertension (x^2^ = 24.5, *p* = 0.001, x^2^ = 12.5, *p* = 0.002 and x^2^ = 12.6, *p* = 0.001, respectively). The adjusted model showed the high TG/HDL-C ratio to be an independent predictor for both a low eGFR and UACR (OR = 1.5, 1.2–1.9 and OR = 1.22, 1.02–1.47, respectively) in combination with old age and hypertension. Conclusion: The TG/HDL-C ratio was revealed to be a potential predictor for both a low eGFR and micro/macroalbuminuria in non-diabetic patients. The arterial stiffening was included in the main underlying pathophysiological mechanisms.

## 1. Highlight

The triglycerides to high-density lipoprotein cholesterol (TG/HDL-C) ratio can predict chronic renal dysfunction in a non-diabetic population of patients defined by both classified eGFR and micro/macroalbuminuria, increasing the risk to 1.5-fold and 1.22-fold, respectively, in combination with older age and hypertension after adjustment for appropriate confounders. Arterial stiffening may be the major underlying mechanism.

## 2. Introduction

In spite of the prevalence rate of chronic kidney disease (CKD) increasing, its causing factors are still incompletely understood. Reduced renal function is related to accelerated atherosclerosis, that is connected to abnormalities in the metabolism of lipids [1,2]. The dyslipidemia in CKD is commonly characterized by the presence of high triglycerides (TG) and low high-density lipoprotein cholesterol (HDL-C) [3].

Serum lipids including elevated total cholesterol, low-density lipoprotein (LDL) and TG or diminished high-density lipoprotein (HDL) have been considered as significant risk factors for CKD [4,5,6]. Changes in the structure and protective features of HDL particles may also occur in CKD including anti-inflammatory and antithrombotic functionalities [7]. Several retrospective and prospective studies have shown that higher apolipoprotein B, which is the main protein of LDL particles and lower apolipoprotein A1, which is the major protein component of HDL, were associated with CKD [8,9]. Another study reported that a higher apolipoproteinB/A1 ratio rather than apolipoprotein B itself was significantly associated with lower estimated glomerular filtration rate (eGFR) [10]. However, no clear superiority of apolipoproteins over traditional lipid measurements in their correlations with CKD has been advocated [10]. Moreover, it has been shown that plasma lipids and apolipoproteins including LDL-cholesterol (LDL-C), triglycerides and apolipoprotein B were associated with elevated urinary albumin excretion [11]. In contrast, a large cohort of patients showed that serum lipids and apolipoproteins were not independently associated with progression of kidney disease [12]. Furthermore, the relationship between dyslipidemia and progression of renal disease is not consistent in studies using diabetic and non-diabetic subjects [13,14]. Therefore, the role of dyslipidemia as a predictor of renal dysfunction is still unclear.

The TG to HDL-C (TG/HDL-C) ratio has been already included in the potential indices for an increased risk of atherosclerotic vascular events in CKD [15], although a few studies considered its role on the chronic renal disease until now.

In this study, we addressed the importance of the TG/HDL-C ratio for the prediction of chronic renal disease defined by both classified eGFR and classified urinary albumin-to-creatinine ratio (UACR) in non-diabetic participants.

## 3. Materials and Methods

### 3.1. Subjects

This is a single-center cross-sectional study, which was conducted in Doctors’ Hospital and it was approved by the Hospital Institutional Review Board. A total of one hundred and eighty-three subjects were included. We collected participants from the Department of Nephrology outpatient clinic of our hospital and they or their legal guardian provided informed oral consent prior to study enrolment.

We studied ninety-seven males and eighty-six females with a mean age of 67.3 ± 15.6 years old after the exclusion of uncooperative patients and those who were younger than eighteen years of age. We did not enclose in the study patients with diabetes mellitus. We also enrolled only non-drinker subjects. Non-drinkers were considered the participants who did not declare any alcohol consumption during the past month. Such a declaration was confirmed by biochemical measurement of transaminases, including alanine aminotransferase (ALT) and aspartate aminotransferase (AST), before the enrollment in the study.

Demographic data regarding age, gender, and lifestyle characteristics including smoking were collected using a questionnaire. Thirty-three of the participants were current smokers (*n* = 33, a ratio equal to 18%).

Detailed individual medical histories and the current pharmaceutical therapy were obtained from the participants. A total of one hundred and thirty-seven participants were hypertensive (*n* = 137, a ratio equal to 74.9%). Every one of the hypertensives was receiving the same anti-hypertensive medications including beta-blockers, calcium channel blockers, and inhibitors of angiotensin II AT1 receptors. Everybody of the enclosed patients was taking the same hypolipidemic medications.

Our staff recorded anthropometric measurements including body weight (to the nearest 0.1 kg) and height (to the nearest 0.1 cm) using an anthropometer (Seca, Hamburg, Germany). BMI was calculated by dividing the body weight in kilograms by the square of the height in meters (kg/m^2^) and categorized based on the WHO classification [16]. Our trained staff also recorded the measurements of waist circumference (WC), which were made approximately at the midpoint between the top of the iliac crest and the lower margin of the last palpable rib at the end of a normal expiration according to the WHO guidelines [17].

### 3.2. Biochemical Measurements

Overnight fasting plasma creatinine (normal range 0.5–1.2 mg/dL), TG (normal range 40–150 mg/dL), LDL-C (normal range < 160 mg/dL) and HDL-C (normal range 35–80 mg/dL) were recorded from the patient files using the latest results. The spectrophotometric technique by the Chemistry Analyzer (MINDRAY BS-200, Diamond Diagnostics, Holliston, MA, USA) was used for biochemical measurements. The TG/HDL-C ratio was calculated. Urinary albumin and creatinine concentrations were measured by the Chemistry Analyzer using spot urine samples from the first morning void.

### 3.3. Definitions

Hypertensives were defined as the subjects who had a mean systolic blood pressure (SBP) ≥ 130 mmHg and/or diastolic blood pressure (DBP) ≥ 85 mmHg and/or those of the participants who were taking antihypertensive therapy due to pre-existing individual history of hypertension according to IDF criteria for metabolic syndrome definition [18]. We requested the measurement of blood pressure at home using an automatic sphygmomanometer Omron M4-I (Omron Co. Ltd., Kyoto, Japan). Every participant doubly measured the blood pressure two times per day, in the morning after rising and in the evening in a fasting, calming and resting state, and they recorded two means per day. Their average was used for statistical analysis. We also used a 24-h ambulatory blood pressure monitor with the Mobil-O-Graph device for verification of measurements. When the mean blood pressure values significantly differed from the home-recorded values, the means of 24-h monitoring were used for statistical analysis rather than the means by home measurements. Peripheral mean blood pressure (pMBP) was calculated as: pMBP = DBP + 0.4 (SBP–DBP). Pulse pressure (PP) was calculated as the difference between SBP and DBP. We also calculated ePWV using the equation described in the study by Greve et al. [19] that was derived by the Reference Values for Arterial Stiffness’ Collaboration [20]. ePWV was calculated from age and MBP: ePWV = 9.587 − 0.402 × age + 4.560 × 10^−3^ × age^2^ − 2.621 × 10^−5^ × age^2^ × MBP + 3.176 × 10^−3^ × age × MBP − 1.832 × 10^−2^ × MBP.

The presence of CKD was defined according to KDIGO 2012 criteria for a duration time more than 3 months [21]. The eGFR was calculated using the CKD Epidemiology Collaboration equation and classified in 5 categories (1 to 5) according to KDIGO 2012 criteria. We also classified our participants in stages based on albuminuria, which was defined as urinary albumin-to-creatinine ratio (ACR) ≥ 30 mg/gr according to KDIGO 2012 [21]. Since UACR correlates well with 24-h urinary albumin excretion, UACR calculation by using a spot urine sample is considered an acceptable calculation [22]. The primary renal disease included hypertensive nephrosclerosis, interstitial nephritis, polycystic nephropathy, and other/unknown.

Central or visceral obesity was determined by a WC ≥ 94 cm in men and ≥80 cm in women using the International Diabetes Federation criteria for diagnosis of metabolic syndrome [18].

## 4. Data Analysis

Data were presented as absolute numbers and frequencies for binary and categorical variables. Data were expressed as mean ± standard deviation or as a median value (interquartile range) for data that showed skewed distribution. The differences between mean values for two groups were assessed by using unpaired *t*-test and data that showed skewed distributions were compared with the Mann–Whitney *U*-test. Bivariate correlations between variables were defined by Spearman coefficients and comparisons between categorical variables were defined by x^2^ tests. Statistically significant was considered a *p*-value < 0.05. We built models using logistic regression analysis by the enter method in order to consider the potential role of the TG/HDL-C ratio on the prediction of both eGFR < 60 mL/min/1.73 m^2^ and UACR > 30 mg/gr, adjusting to age, gender, BMI, smoking, and hypertension. Power calculation was performed at the end of the study using the Power and Precision statistical package (version 3.0, Biostat, Englewood, NJ, USA).

## 5. Results

The characteristics of the studied population are shown in Table 1.

### 5.1. Correlations

Bivariate correlations between the TG/HDL-C ratio and variables are depicted in Table 2. We observed a significantly positive correlation of the TG/HDL-C ratio with BMI, WC, LDL-C, blood pressure, ePWV, and UACR, although the correlation with eGFR was found to be significantly opposite.

### 5.2. Comparisons

We divided our patients in two groups according to the TG/HDL-C ratio cut-off point, either with an eGFR value of less or more than 60 mL/min/1.73 m^2^, equal to 3.41 (greater, *n* = 81 or lower, *n* = 102 than 3.41). Characteristics and differences between the two groups of patients are listed in Table 3.

**Figure 1:**
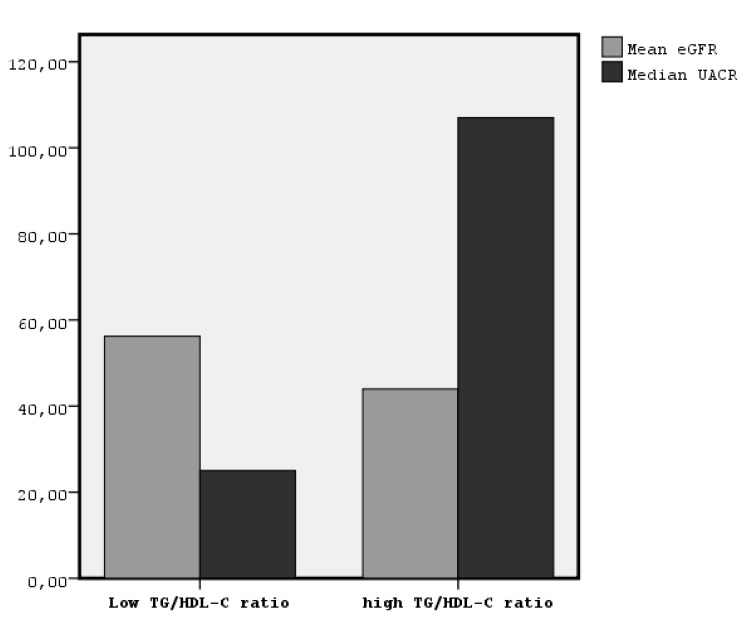
The comparison between the high and low TG/HDL-C ratio regarding both eGFR and UACR (mean ± SD 43.9 ± 20 vs. 58.8 ± 22.1 (*p* = 0.001) and mean rank 107.1 vs. 80.0 (*p* = 0.001), respectively).

The comparison between the high and low TG/HDL-C ratio showed the following: age mean ± SD 70.1 ± 14.8 vs. 65.1 ± 15.9 (*p* = 0.03), LDL-C 1 mean ± SD 19.7 ± 19.2 vs. 109.3 ± 29.7 (*p* = 0.005), TG mean rank 136.7 vs. 54.8 (*p* = 0.001), HDL-C mean rank 49.9 vs. 125.4 (*p* = 0.001), BMI mean rank 108.8 vs. 78.6 (*p* = 0.001), WC mean rank 105.8 vs. 81.01 (*p* = 0.002), ePWV mean ± SD 12.4 ± 2.6 vs. 11.3 ± 2.7 (*p* = 0.006), SBP mean rank 107.8 vs. 79.4 (*p* = 0.001), pMBP mean rank 107.1 vs. 80.0 (*p* = 0.001), and PP mean ± SD 67.02 ± 14.3 vs. 60.06 ± 16.7 (*p* = 0.003). The comparison between the high and low TG/HDL-C ratio regarding both eGFR and UACR is depicted in Figure 1 (mean ± SD 43.9 ± 20 vs. 58.8 ± 22.1 (*p* = 0.001) and mean rank 107.1 vs. 80.0 (*p* = 0.001), respectively).

### 5.3. Categorical Associations

**Figure 2:**
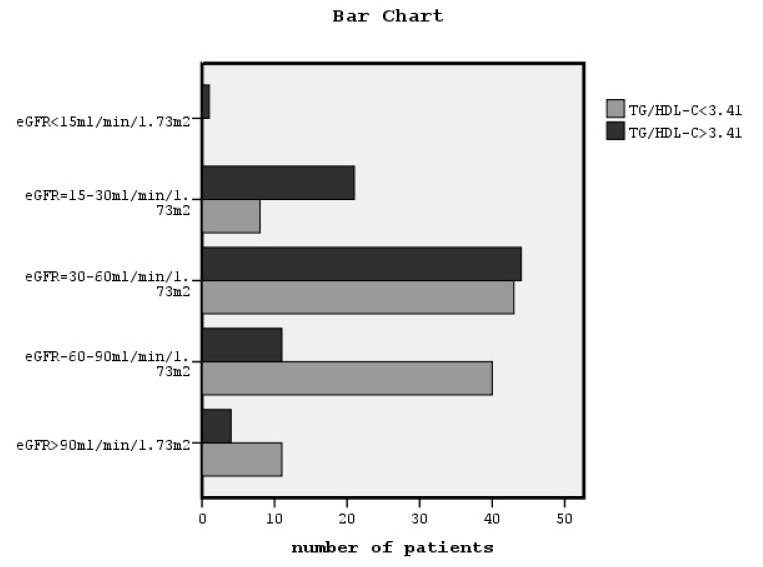
The association between TG/HDL-C ratio greater or lower than the cut-off point related to an eGFR of less than 60 mL/min/1.73 m^2^ and classified eGFR (x^2^ = 24.5, *p* = 0.001).

**Figure 3:**
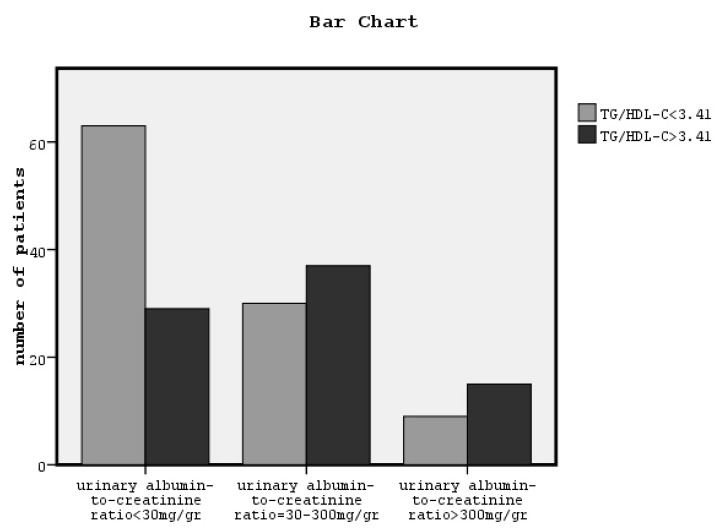
The association between TG/HDL-C ratio greater or lower than the cut-off point related to an eGFR of less than 60 mL/min/1.73 m^2^ and classified urinary albumin excretion (x^2^ = 12.5, *p* = 0.002).

X^2^ tests showed a significant association between a high TG/HDL-C ratio and classified eGFR, and classified UACR and hypertension (x^2^ = 24.5, *p* = 0.001, Figure 2, x^2^ = 12.5, *p* = 0.002, Figure 3 and x^2^ = 12.6, *p* = 0.001, respectively). The relationship between TG/HDL-C ratio and both gender and smoking was found to be non-significant.

### 5.4. Adjusted Models

The built model using logistic regression analysis showed the high TG/HDL-C ratio to be a significant independent predictor, increasing the risk to 1.5-fold (OR = 1.5, 1.2–1.9) for a low eGFR in combination with old age (OR = 1.07, 1.04–1.11) and hypertension (OR = 3.08, 1.2–7.7) after adjustment to covariates including gender, BMI, and smoking (Table 4). We also observed the high TG/HDL-C ratio to be a potential risk factor for UACR combined with old age (OR = 1.22, 1.02–1.47 and OR = 1.03, 1.01–1.06, respectively) adjusting to the same covariates (Table 5). The power calculation of the above logistic analyses showed that our sample size of 183 patients provides a power of 90% at a two-tailed α = 0.05 to detect a significant association between these variables.

## 6. Discussion

In this study, for the first time to our knowledge, we showed that a high TG/HDL-C ratio can predict chronic renal dysfunction in non-diabetic patients defined by both classified eGFR and micro/macroalbuminuria, increasing the risk to 1.5-fold and 1.22-folds, respectively, in combination with older age and hypertension after adjustment for appropriate confounders. We observed that in the group of patients with a high TG/HDL-C ratio, most patients were in the third and fourth eGFR stage, although in the low TG/HDL-C ratio group of patients, most patients were in the first, second, and third eGFR stage and none were in the fifth eGFR stage, as it is depicted in Figure 1. Similarly, most patients with a low TG/HDL-C ratio had neither micro- or macroalbuminuria, as it is depicted in Figure 2. Moreover, in our data, the group of patients with a higher TG/HDL-C ratio were older and they had significantly higher LDL-C, BMI, central obesity, blood pressure, arterial stiffness, and albuminuria, although they had significantly lower eGFR than the group of patients with a lower TG/HDL-C ratio. Moreover, the bivariate correlations between TG/HDL-C ratio and the above variables were found to be statistically significant.

In some agreement with our findings, one previous retrospective study reported a relationship of TG/HDL-C ratio with development of CKD with a hazard ratio equal to 1.22 (1.12–1.32) considering only an eGFR < 60 mL/min/1.73 m^2^, as the unique index of CKD, during a mean follow-up of 56.5 ± 14.3 months in apparently healthy subjects [6]. It has been also reported that high TG and low HDL-C predict an increased risk of renal dysfunction considering serum creatinine as an index of renal function [4]. On the other hand, it has been previously shown that increased urinary albumin excretion was significantly associated with lipids disorders including TG adjusting for sex and age with minor changes being observed between micro- and macroalbuminuria [11]. In discrepancy, another previous study did not reveal any significant association between lipids/lipoproteins abnormalities and progression of kidney disease [12].

Atherosclerosis of the small and medium-sized vessels is a potential predictor for decline of renal function with progressive age [23,24]. The association of lipids and lipoproteins with kidney function is partially mediated by the effects of these lipoproteins on atherogenesis [25]. Hypertension, diabetes mellitus, and obesity are common risk factors for atherosclerosis and influence the serum levels of lipoproteins. In this study, we excluded participants with diabetes mellitus and we tried to minimize confounding by the other two factors through adjustment in statistical analysis.

Apolipoprotein B, which is a component of LDL-C and TG, contributes to atherogenesis, although the protective role of HDL-C, which mainly includes apolipoprotein A1, on atherosclerosis has been described [26,27]. In our study, we did not consider apolipoproteins B or A1 since many previous studies have reported on them and also a clear superiority of apolipoproteins over traditional lipids in their association with eGFR or CKD has not been suggested [10]. Recently, a TG/HDL-C ratio was comprised of the potential risk factors for atherosclerotic vascular events in CKD [15]. In agreement, in our study we noted a significant unadjusted association of a high TG/HDL-C ratio with manifested hypertension and the patients with a higher TG/HDL-C ratio had significantly higher blood pressure and arterial stiffening in comparison to the group of patients with a lower TG/HDL-C ratio.

Additional mechanisms beyond atherosclerosis of major vessels may be involved in the relationship of lipoproteins with renal disease. These may include direct toxic effects of lipids on glomerular cells, such as podocytes [28,29]. The lower levels of the antioxidative apolipoprotein A1-enriched HDL in CKD accelerate oxidative stress, resulting in a toxic effect on glomeruli. Oxidative stress contributes in increased oxidized-apolipoprotein B-enriched LDL, local formation of foam cells, and activation of inflammation [5]. The lower levels of apolipoprotein A1-enriched HDL during CKD have also been combined with reduced anti-inflammatory effects [30]. In the meantime, changes in the anti-inflammatory and antithrombotic functionalities of HDL particles in CKD have been supported [7].

On the other hand, abnormalities in the metabolism of lipoproteins, such as increased LDL production, reduced LDL clearance, and elevated TG are closely connected to urinary albumin excretion [31,32]. It has been established that albuminuria is not only an early marker of renal disease, but it also is considered a marker of generalized endothelial damage caused partly by retention of apolipoprotein B-enriched lipoproteins in the arterial wall [33].

Furthermore, previously it has been reported that an elevated TG/HDL-C ratio was significantly associated with increased insulin resistance in apparently healthy subjects [34]. Recently, it has been suggested that the serum log TG/HDL-C ratio was the most suitable predictor of CKD, and insulin resistance may be the causing mechanism in subjects without known CKD or renal impairment [35]. In the meantime, insulin resistance is common in CKD due to increased oxidative stress and the uremic inflammatory environment [36].

According to our findings, we could suggest that arterial stiffening and hypertension in progressive age may be the major underlying mechanisms by which a high TG/HDL-C ratio can contribute and predict the decline of renal function in a non-diabetic population of patients.

## 7. Limitations

The main limitation of this study is the cross-sectional nature in the Department of Nephrology of one single-center, which does not allow the establishment of cause–effect relationships.

## 8. Conclusions

In this study, we showed the TG/HDL-C ratio to be a potential independent risk factor for chronic renal disease defined by both a low eGFR and micro/macroalbuminuria in non-diabetic patients. The arterial stiffening and hypertension in progressive age were found to be the main underlying pathophysiological mechanisms.

## Figures and Tables

**Table 1:** Characteristics in the entire cohort (*n* = 183).

Characteristic	Mean/Median	SD/Interquartile Range
Age (years)	67.3	15.6
BMI (Kg/m^2^)	28	26–31
WC (cm)	101	95–110
LDL-C (mg/dL)	113.9	26.05
TG/HDL-C	3.37	1.9
SBP (mmHg)	146	130–155
DBP (mmHg)	80	75–90
MBP (mmHg)	107.6	99.2–112
ePWV (m/s)	11.77	2.7
PP (mmHg)	63.14	16.03
UACR (mg/gr)	28.7	11.2–125
eGFR (ml/min/1.73 m^2^)	52.2	22.4
Category Variables	*n (%)*	
Gender (males/females)	97 (53%)/86 (47%)	
Hypertension (yes/no)	137(74.9%)/46(25.1%)	
Smoking (yes/no)	33 (18%)/150 (82%)	
Anti-hypertensive medications (yes/no)- beta-blockers - calcium channel blockers - inhibitors of angiotensin II AT1 receptors	137(74.9%)/46(25.1%)	
Primary renal disease		
- hypertensive nephrosclerosis	115 (62.8%)	
- interstitial nephritis	31 (16.9%)	
- polycystic nephropathy	4 (2.2%)	
- other/unknown	33 (18%)	

BMI: body mass index; WC: waist circumference; LDL-C: low-density lipoprotein cholesterol; TG/HDL-C: triglycerides to high-density lipoprotein cholesterol; SBP: systolic blood pressure; DBP: diastolic blood pressure; MBP; mean blood pressure; ePWV: estimated pulse wave velocity; PP: pulse pressure; UACR: urinary albumin-to-creatinine ratio; eGFR: estimated glomerular filtration rate.

**Table 2:** Correlation of TG/HDL-C ratio with different studied variables in our data (*n* = 183).

Variables	TG/HDL-C	
	*r*	*p* value
Age (years)	0.116	0.1
BMI (Kg/m^2^)	0.344	0.001
WC (cm)	0.302	0.001
LDL-C (mg/dL)	0.306	0.001
SBP (mmHg)	0.311	0.001
DBP (mmHg)	0.145	0.05
MBP (mmHg)	0.325	0.001
ePWV (m/s)	0.177	0.01
PP (mmHg)	0.245	0.001
eGFR (ml/min/1.73 m^2^)	−0.336	0.001
UACR (mg/gr)	0.280	0.001

*r*: Spearman correlation coefficient; TG/HDL-C: triglycerides to high-density lipoprotein cholesterol; BMI: body mass index; WC: waist circumference; LDL-C: low-density lipoprotein cholesterol; SBP: systolic blood pressure; DBP: diastolic blood pressure; MBP; mean blood pressure; ePWV: estimated pulse wave velocity; PP: pulse pressure; eGFR: estimated glomerular filtration rate; UACR: urinary albumin-to-creatinine ratio; *p* value < 0.05 is significant.

**Table 3:** The differences between groups of patients according to the TG/HDL-C ratio cut-off point, either with an eGFR of less or more than 60 mL/min/1.73 m^2^, equal to 3.41.

Characteristic	Patients with TG/HDL-C > 3.41 (*n* = 81) mean ± SD	Patients with TG/HDL-C < 3.41 (*n* = 102) mean ± SD	*p* Value
Age (years)	70.1 ± 14.8 *	65.1 ± 15.9	0.03
BMI (Kg/m^2^)	Mean Rank = 108.8 *	78.7	0.001
WC (cm)	Mean Rank = 105.8 *	81.01	0.002
LDL-C (mg/dL)	119.7 ± 19.2 *	109.3 ± 29.7	0.005
TG/HDL-C	4.9 ± 1.7 *	2.1 ± 0.7	0.001
SBP (mmHg)	Mean Rank = 107.8 *	79.4	0.001
DBP (mmHg)	Mean Rank = 97.07	87.9	0.2
MBP (mmHg)	Mean Rank = 107.1 *	80.0	0.001
ePWV (m/s)	12.3 ± 2.6 *	11.3 ± 2.7	0.006
PP (mmHg)	67.02 ± 14.3 *	60.06 ± 16.7	0.003
UACR (mg/gr)	Mean Rank = 107.2 *	80.1	0.001
eGFR (ml/min/1.73 m^2^)	43.9 ± 20.0 *	58.8 ± 22.1	0.001
Category variables	*n* (%)	*n* (%)	
Hypertension (yes/no)	71(51.8%)/10(21.7%) *	66(48.2%)/36 (78.3%)	0.001
Smoking (yes/no)	17 (21%)/64 (79%) *	16 (15.7%)/86(84.3%)	0.2

TG/HDL-C: triglycerides to high-density lipoprotein cholesterol; BMI: body mass index; WC: waist circumference; LDL-C: low-density lipoprotein cholesterol; SBP: systolic blood pressure; DBP: diastolic blood pressure; MBP; mean blood pressure; ePWV: estimated pulse wave velocity; PP: pulse pressure; eGFR: estimated glomerular filtration rate; UACR: urinary albumin-to-creatinine ratio; *: *p* < 0.05.

**Table 4:** Logistic regression analysis for the prediction of eGFR < 60 mL/min/1.73 m^2.^

Variables in Model	p-value	Odds Ratio	Confidence Interval
Age (years)	0.001	1.07	1.04–1.11
Gender (males/females)	0.09	0.5	0.2–1.1
BMI (Kg/m^2^)	0.7	1.01	0.9–1.09
Hypertension (yes/no)	0.01	3.08	1.2–7.7
Smoking (yes/no)	0.2	0.6	0.2–1.5
TG/HDL-C	0.001	1.5	1.2–1.9

eGFR: estimated glomerular filtration rate; BMI: body mass index; TG/HDL-C: triglycerides to high-density lipoprotein cholesterol.

**Table 5:** Logistic regression analysis for the prediction of UACR > 30 mg/gr.

*Variables in Model*	*p*-value	Odds Ratio	Confidence Interval
Age (years)	0.002	1.03	1.01–1.06
Gender (males/females)	0.4	0.7	0.4–1.5
BMI (Kg/m^2^)	0.9	1.002	0.9–1.08
Hypertension (yes/no)	0.08	2.1	0.9–4.9
Smoking (yes/no)	0.9	1.04	0.5–2.4
TG/HDL-C	0.03	1.22	1.02–1.47

UACR: urinary albumin-to-creatinine ratio; BMI: body mass index; TG/HDL-C: triglycerides to high-density lipoprotein cholesterol.
